# The circulatory small non‐coding RNA landscape in community‐acquired pneumonia on intensive care unit admission

**DOI:** 10.1111/jcmm.16406

**Published:** 2021-07-17

**Authors:** Hina N. Khan, Aldo Jongejan, Lonneke A. van Vught, Janneke Horn, Marcus J. Schultz, Aeilko H. Zwinderman, Olaf L. Cremer, Marc J. Bonten, Tom van der Poll, Brendon P. Scicluna

**Affiliations:** ^1^ Center for Experimental Molecular Medicine Amsterdam University Medical Centers location Academic Medical Center University of Amsterdam The Netherlands; ^2^ Department of Clinical Epidemiology, Biostatistics and Bioinformatics Amsterdam University Medical Centers location Academic Medical Center Amsterdam The Netherlands; ^3^ Department of Intensive Care & Laboratory of Experimental Intensive Care and Anesthesiology (L·E·I·C·A) Amsterdam University Medical Centers location Academic Medical Center Amsterdam The Netherlands; ^4^ Mahidol‐Oxford Tropical Medicine Research Unit (MORU) Mahidol University Bangkok Thailand; ^5^ Department of Intensive Care University Medical Center Utrecht Utrecht The Netherlands; ^6^ Department of Medical Microbiology University Medical Center Utrecht Utrecht The Netherlands; ^7^ Julius Center for Health Sciences and Primary Care University Medical Center Utrecht Utrecht The Netherlands; ^8^ Division of Infectious Diseases Amsterdam University Medical Centers location Academic Medical Center University of Amsterdam Amsterdam The Netherlands

**Keywords:** community‐acquired pneumonia, micro RNA, Sepsis, small non‐coding RNA, *Streptococcus pneumoniae*

## Abstract

Community‐acquired pneumonia (CAP) is a major cause of sepsis. Despite several clinical trials targeting components of the inflammatory response, no specific treatment other than antimicrobial therapy has been approved. This argued for a deeper understanding of sepsis immunopathology, in particular factors that can modulate the host response. Small non‐coding RNA, for example, micro (mi)RNA, have been established as important modifiers of cellular phenotypes. Notably, miRNAs are not exclusive to the intracellular milieu but have also been detected extracellular in the circulation with functional consequences. Here, we sought to determine shifts in circulatory small RNA levels of critically ill patients with CAP‐associated sepsis and to determine the influence of clinical severity and causal pathogens on small RNA levels. Blood plasma was collected from 13 critically ill patients with sepsis caused by CAP on intensive care unit admission and from 5 non‐infectious control participants. Plasma small RNA‐sequencing identified significantly altered levels of primarily mature miRNAs in CAP relative to controls. Pathways analysis of high or low abundance miRNA identified various over‐represented cellular biological pathways. Analysis of small RNA levels against common clinical severity and inflammatory parameters indices showed direct and indirect correlations. Additionally, variance of plasma small RNA levels in CAP patients may be explained, at least in part, by differences in causal pathogens. Small nuclear RNA levels were specifically altered in CAP due to Influenza infection in contrast to *Streptococcus pneumoniae* infection. Pathway analysis of plasma miRNA signatures unique to Influenza or *Streptococcus pneumoniae* infections showed enrichment for specific proteoglycan, cell cycle, and immunometabolic pathways.

## 1 INTRODUCTION

Community‐acquired pneumonia (CAP) is a major determinant of sepsis, which accounts for substantial morbidity and mortality worldwide.^1^ Sepsis is understood to be mainly initiated by a severe, and often protracted, reaction to infection leading to organ failure.^2^ The aetiology of sepsis is multifaceted, involving various microbiological and host response variables.^3^ The early oversimplified model of an overwhelming inflammatory reaction failed to capture the complex pathophysiology of the sepsis syndrome, exemplified by unsuccessful clinical trials with a variety of anti‐inflammatory agents.^4^ New insights have suggested that while inflammation is responsible for ‘collateral’ tissue damage, immune suppression that accompanies sepsis also contributes to adverse clinical outcome.^5^ Multiple circulatory factors, for example, soluble mediators of inflammation, can mediate cellular immune suppression via autocrine and/or paracrine signalling activity. In addition, circulating nucleic acids (CNAs) can alter cellular phenotypes by specific interference with gene expression,^6^ demonstrated by micro (mi)RNA mediated post‐transcriptional gene regulation.^7^ Levels of CNAs have been shown to be altered in serum of sepsis patients.^8^ Knowledge of CNA levels in plasma of sepsis patients with CAP, particularly small non‐coding RNA, and the influence of clinical severity and causal pathogens on those profiles is lacking.

CNAs are segments of a host's or pathogen's genome present in biological fluids and have been known to exist for decades.^9^ These molecules are understood to be released in the host's circulation via either passive secretion, for example, cell death, or active secretion mechanisms.^7^ CNAs have been found in the circulation of non‐infectious control participants and also shown to be significantly altered in disease conditions, such as cancer,^10^ trauma ^11^ and autoimmune disorders.^12^ Such observations have suggested that CNAs may represent promising, non‐invasive, tools for early detection of several diseases.

In this study, we sought to determine changes in CNAs, specifically small non‐coding RNAs, measured in blood plasma samples from patients with CAP‐associated sepsis on intensive care unit (ICU) admission. Levels of plasma small RNAs in CAP were firstly compared to non‐infectious control participants, as well as patients with blood culture positive *Streptococcus (S.) pneumoniae* compared to patients with polymerase chain reaction (PCR) positive Influenza (A or B) infections. Significantly different plasma miRNA levels were analysed for biological pathway enrichment, revealing putative functional targets of paracrine and/or autocrine signalling.

## 2 METHODS

### 2.1 Study design

The study was performed within the context of the Molecular Diagnosis and Risk Stratification of Sepsis (MARS) project, a prospective observational cohort study in the two tertiary referral centres in the Netherlands (ClinicalTrials.gov identifier NCT01905033) (Academic Medical center, Amsterdam and University Medical Center, Utrecht).^13^, ^14^ The current study comprised a total of 13 ICU‐admitted CAP patients with positive blood cultures for *S pneumoniae* and PCR positive Influenza A or B infection. Sepsis diagnosis was described in detail previously.^13^, ^15^ This cohort was enrolled between January 2011 and July 2012. The Medical Ethics committees approved an opt‐out consent method (IRB no. 10‐056C). For each patient, demographics, comorbidities, severity indices and outcomes were gathered. Severity was assessed by APACHE IV^16^ and mSOFA excluding the central nervous system component.^17^ Septic shock was defined by the use of noradrenaline for hypotension in a dose of more than 0.1µg/kg/min during at least 50% of the ICU day. Five non‐infectious control participants (age and gender‐matched) were also included in the study. From all non‐infectious control participants’ written informed consent was obtained. Procedures were performed in accordance with the Helsinki declaration of 1975 (revised 1983). Using 13 sepsis patient samples and 5 non‐infectious control participants, and estimated effect size of 0.8, we had 80% power to reject the null hypothesis (5% false discovery rate).

### 2.2 Sampling, small RNA isolation and sequencing

Whole blood (EDTA) collected from non‐infectious control participants and sepsis patients were centrifuged at 1200rpm for 20 minutes to obtain 300ul plasma, and stored in −80C. Plasma from sepsis patients was obtained within 6 hours of ICU admission. Prior to nucleic acid isolation, plasma samples were thawed over ice and centrifuged at 12,000rpm for 10 minutes. Small non‐coding RNA was isolated from plasma samples using the miRNeasy mini kit (Qiagen) and MinElute PCR purification kit (Qiagen) as described by the manufacturer. Next generation sequencing libraries of small RNA were prepared using the Illumina TruSeq Small RNA‐Seq sample preparation kit (Illumina). Small non‐coding RNA was isolated from plasma samples using the miRNeasy mini kit (Qiagen) and MinElute PCR purification kit (Qiagen) as described by the manufacturer. Quantity and quality of isolated small RNA were evaluated by means of bioanalysis (Agilent 2100 Bioanalyzer) and Agilent RNA 6000 Pico chip kit (Agilent) according to manufacturer's specifications. Next generation sequencing libraries of small RNA were prepared using the Illumina TruSeq Small RNA‐Seq sample preparation kit (Illumina). After adaptor ligation and size selection, the excised product was used for PCR amplification with quality and yield measured with Agilent High Sensitivity DNA Chips (Agilent) in an Agilent 2100 bioanalyzer. Clustering and cDNA sequencing was performed using the Illumina cBot and HiSeq 2500 (Illumina). Library preparation and sequencing were done at GenomeScan, Leiden, the Netherlands (www.genomescan.nl) using Solexa sequencing technology (Illumina) as described by manufacturer.

### 2.3 Bioinformatics of small RNA‐sequencing

Quality of raw sequencing data (.fastq files) was assessed using FastQC v0.11.5 (http://www.bioinformatics.babraham.ac.uk/projects/fastqc/). Trimmomatic version 0.32^18^ was used to trim the Illumina adapters, poor‐quality bases and ambiguous nucleotide‐containing sequences. Low quality leading (3 nucleotides) and trailing bases (3 nucleotides) were removed from each read, a sliding window trimming using a window of 4 bases and clipped the read once the average quality within the window falls below a threshold of 15 and dropped reads if below 18 nucleotides. After pre‐processing, the remaining high‐quality reads were used to align against the Genome Reference Consortium Human Build 38 patch release 7 (GRCh38.p7),^19^ using Tophat2 version 2.1.1^20^ with default parameters. The featureCounts v1.5.0‐p1 program^21^ was used to quantify the abundance of all species of small non‐coding RNA. The Bioconductor package DESeq2 (version 1.14.1)^22^ was used for differential expression analysis using a detection threshold greater than 2 counts. Throughout Benjamini‐Hochberg (BH)^23^ multiple comparison‐adjusted probabilities (adjusted *P* <.05) and fold change ≥ 1.5 or ≤ −1.5 defined significance. After annotation with featureCounts v1.5.0‐p1, filtered out genes where there were counts greater than or equal to 10 in at least one sample. MirPath v3.0^24^ was used for the identification of cellular biological pathways, which utilize predicted targets from TarBase v7.0^25^ having more than 600,000 experimentally supported interactions. Human species annotations and 3’UTR interactions were specified. Significant pathways were defined by adjusted Benjamini‐Hochberg's (BH) p‐values < 0.05.

### 2.4 Statistics

Statistical analysis was performed in the R statistical environment (v 3.5.0; R Core Team (2019). R: A language and environment for statistical computing. R Foundation for Statistical Computing, Vienna, Austria. URL https://www.R‐project.org/.). For correlation studies, we used the Spearman correlation method. The Hmisc R package was used to compute the significance levels for spearman correlations and corrplot R package was used to create the correlogram. Heat maps were generated using the pheatmap R method and principal component analysis (PCA) was done using the FactoMinerR R package. Venn diagrams were made using the VennDiagram R package.

## 3 RESULTS

### 3.1 Circulatory small non‐coding RNA repertoire in CAP relative to controls

**FIGURE 1:**
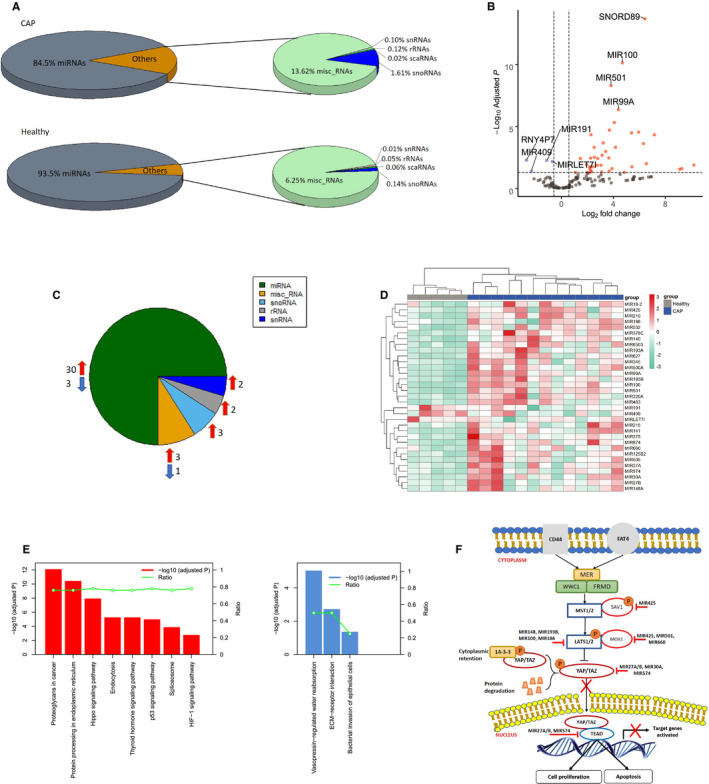
Landscape of small non‐coding RNA species in patients with sepsis caused by community‐acquired pneumonia (CAP) and controls. (**A**) Pie charts showing the proportion of reads aligning uniquely to small non‐coding RNAs (averaged across all samples). The vast majority of reads aligned to miRNAs. (**B**) Volcano plot showing log2‐transformed fold change and Benjamini‐Hochberg (BH) adjusted p‐values (‐log 10 transformed). 44 significantly altered small RNA species were detected. Red dots denote significantly elevated small RNA molecules; blue dots depict significantly reduced small RNAs. (**C**) Pie chart illustrating various small RNA species in CAP‐associated sepsis relative to controls. miRNA, micro RNA; misc_RNA, miscellaneous RNA; snoRNA, small nucleolar RNA; rRNA, ribosomal RNA; snRNA, small nuclear RNA. Red or blue arrows depict high or low abundance patterns, respectively. (**D**) Heatmap of the 33 significantly miRNAs (BH adjusted p‐value < 0.05). Columns represent samples and rows represent miRNA expression indices. Red, high abundance; blue, low abundance. (**E**) Pathway analysis of the list of differentially abundant miRNA split as high (red) or low (blue) abundance in patients relative to controls. Ratio (right vertical axis) represents the numbers of miRNAs in a given pathway divided by the total number of miRNAs. (**F**) Schematic diagram of the experimentally observed miRNA‐to‐gene interaction networks involved in Hippo signalling pathway

RNA‐sequencing data of blood plasma from 5 non‐infectious control participants (median age [Q1‐Q3]: 51 [48‐54]; males [%]: 3 [60]) and 13 CAP patients were used to map circulatory small non‐coding RNA. Table 1 shows the baseline characteristics of CAP patients that consisted of adults (median age [Q1‐Q3]: 59 [46‐66]; males [%]: 6 [60]) with median Acute Physiology and Chronic Health Evaluation IV (APACHE IV) score of 79 (Q1‐Q3: 71‐110), median Sequential Organ Failure Assessment (SOFA) score equating to 9 (Q1‐Q3: 7‐10)) and 8 patients (62%) had septic shock; 2 patients did not survive, as assessed after 28‐days of ICU admission. The causative pathogens were blood culture positive *S pneumoniae* infection (n = 8, 62%) or PCR positive Influenza A or B infection (n = 5, 38%). After pre‐processing, read quality filtering and alignment to the human genome, 180k and 695k unique reads in CAP and non‐infectious control participants were available for further analysis. In CAP, the proportion of reads that uniquely aligned to all different non‐coding RNA species showed preponderance of miRNAs (84.5%). Other small non‐coding RNA species included miscellaneous RNA (misc_RNA: 13.62%), small nucleolar RNA (snoRNA: 1.61%), ribosomal RNA (rRNA: 0.12%), small nuclear RNA (snRNA: 0.10%) and small cajal body RNA (scaRNA: 0.02%) (Figure 1A). In non‐infectious control participants, the most abundant species of small RNAs were also miRNA (93.5%) followed by misc_RNA (6.25%), snoRNA (0.14%), rRNA (0.05%), snRNA (0.01%) and scaRNA (0.06%).

**TABLE 1:** Patient demographics and clinical characteristics

	Community‐acquired pneumonia (CAP) n(13)
Demographics	
Age, median (IQR)	59 (46, 66)
Gender male, n *(%)*	6 (60%)
Cormorbidities, n*(%)*	
COPD	2 (15%)
Diabetes_mellitus	3 (23%)
Hypertension	5 (39%)
Congestive_heart_failure	0 (0%)
Causative Pathogen	
*Streptococcus pneumoniae*	8 (62%)
Influenza virus	5 (38%)
Severity indices	
APACHE_IV_Score, median (IQR)	79 (71, 110)
SOFA Score,median (IQR)	9 (7,10)
Septic shock, n(%)	8 (62%)
Outcomes, n(%)	
Mortality_28d	2 (15%)

Note

Value are given as numbers (%) or median [interquartile range].

Abbreviations: APACHE IV = Acute Physiology and Chronic Health Evaluation IV score; COPD = chronic obstructive pulmonary disease; IQR = interquartile rangeSOFA = Sequential Organ Failure Assessment.

Comparing CAP patients to non‐infectious control participants uncovered 44 significantly altered plasma small non‐coding RNA (Figure 1B). The largest class were miRNA (n = 33), followed by misc_RNA (n = 4), snoRNA (n = 3), snRNA (n = 2) and rRNA (n = 2) (Figure 1C and Table S1). MIR100, MIR501, MIR99A, MIR483, MIR141, MIR378C and MIR193B were among the most elevated plasma miRNAs in CAP, whereas MIR191, MIR409 and MIRLET7I were reduced (Figure 1D). Pathway analysis of the high abundance miRNA revealed significant over‐representation for proteoglycans in cancer, hippo signalling pathway, protein processing in endoplasmic reticulum, endocytosis as well as known metabolic pathways, for example, p53 signalling and hypoxia‐inducible factor (HIF)‐1 signalling (Figure 1E). Low abundance circulatory microRNAs were associated with ECM‐receptor interaction, bacterial invasion of epithelial cells and vasopressin‐regulated water reabsorption. Considering adjusted p‐values and highest enrichment ratios, the hippo signalling pathway was uncovered as top over‐represented pathway (Figure 1F).

### 3.2 Circulatory small RNA levels, clinical severity, inflammatory parameters and causative pathogens

**FIGURE 2:**
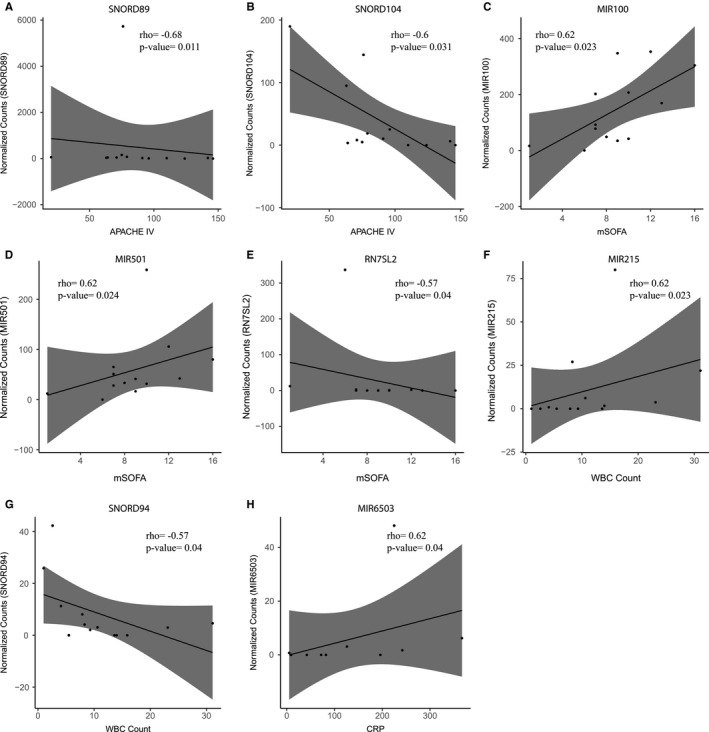
Correlation analysis of circulatory small non‐coding RNA expression against clinical severity indices of patients. Dot plots of (**A**) and (**B**) SNORD104 plasma levels against Acute Physiology and Chronic Health Evaluation (APACHE) IV scores. (**C‐E**) Dot plots illustrating the correlation of (**C**) MIR100, (**D**) MIR501 and (**E**) RN7SL2 plasma levels against modified Sequential Organ Failure Assessment (mSOFA) scores (excluding the central nervous system component). rho, Spearman's coefficient. (**F‐G**) Dot plots illustrating the correlation of (**F**) MIR215 and (**G**) SNORD94 plasma levels white blood cells (WBC) counts. **(H)** Dot plots illustrating the correlation of MIR6503 plasma levels against C‐reactive protein

Here, we first sought to define small RNA levels associated with clinical severity indices and inflammatory parameters and secondly, determine alterations in plasma small RNA dependent on causative pathogen in CAP. Correlation analysis of those significantly altered plasma small RNAs in CAP patients (relative to controls) against APACHE IV scores uncovered two significant inverse correlations (Figure. S1), specifically SNORD89 (Spearman's rho = −0.68, *P* =.011) (Figure 2A) and SNORD104 (rho = −0.6, *P* =.031) (Figure 2B). Significant correlations were also detected against modified (m)SOFA scores (Figure. S1), including MIR100 (rho = 0.62, *P* =.023), MIR501 (rho = 0.62, *P* =.024) (Figure 2C‐D) and RN7SL2 (rho = −0.57, *P* =.04) (Figure 2E). We also evaluated the white blood cell (WBC) count, C‐reactive protein (CRP) and lactate levels (Figure 2F‐H). Two significant correlations were revealed against WBC count, including MIR215 (rho = 0.62, *P* =.023) and SNORD94 (rho = −0.57, *P* =.04) (Figure 2F‐G) and one significant correlation against CRP, MIR6503 (rho = 0.62, *P* =.04) (Figure 2H). No correlation was found for lactate levels. This suggests that inter‐individual variation of plasma small non‐coding RNA levels in CAP patients can be explained, at least in part, by differences in clinical severity and inflammatory variables.

**FIGURE 3:**
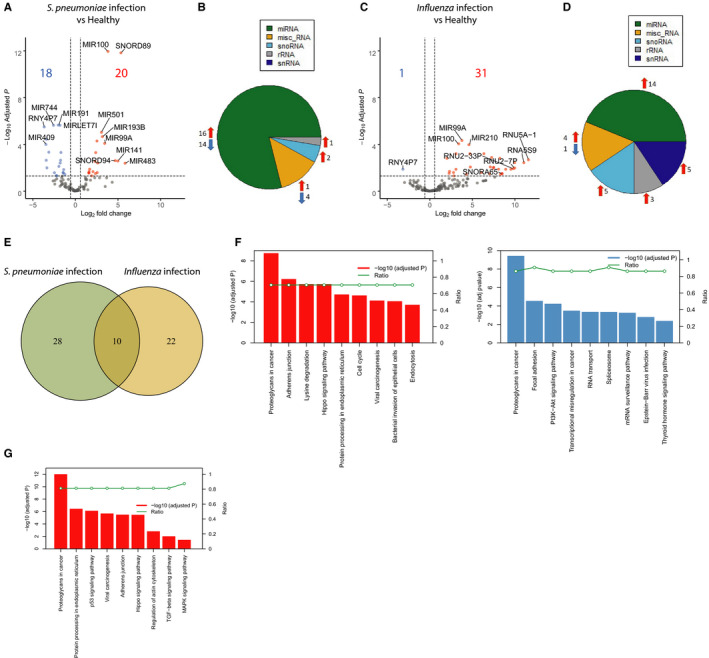
Common and unique small non‐coding RNA levels in patients with sepsis caused by community‐acquired pneumonia (CAP) having either *Streptococcus pneumoniae* (*S pneumoniae*) blood culture positive infection or Influenza (A and/or B) PCR positive infection. (**A**) Volcano plot representing the differentially abundance of small non‐coding RNA in *S pneumoniae* infected patients relative to controls. Horizontal dotted line represents the Benjamini‐Hochberg (BH) adjusted p‐value ≤ 0.05 threshold. Red dots, significant‐high abundance; blue dots, significant low abundance; grey dots, not altered. (**B**) Pie chart illustrating the different small non‐coding RNA species in *S pneumoniae* infection relative to controls. miRNA, micro RNA; misc_RNA, miscellaneous RNA; snoRNA, small nucleolar RNA; rRNA, ribosomal RNA; snRNA, small nuclear RNA. (**C**) Volcano plot depicting small non‐coding RNA changes in plasma of patients with influenza infection as compared to controls. (**D**) Pie chart of small non‐coding RNA species in plasma of patients with influenza infection. (**E**) Venn diagram illustrating common and unique small non‐coding RNAs between *S pneumoniae* and influenza*‐*infected patients. (**F**) Bar graph depicting pathway analysis of the unique miRNA in *S pneumoniae* infection. Red bars, pathways of high abundance miRNA; blue bars, low abundance miRNA pathways. Ratio (right vertical axis) represents the numbers of miRNAs in a given pathway divided by the total number of miRNAs. (**G**) Bar graph showing significantly enriched pathways of the unique high abundance miRNA in patients with influenza infection

In order to evaluate the influence of divergent causative pathogens in CAP on plasma small RNA levels, we grouped CAP patients with either blood culture positive *S pneumoniae* or Influenza PCR positive (A or B) infection. Considering adjusted p‐values < 0.05 and fold change thresholds (≥ 1.5 or ≤ −1.5), *S pneumoniae* infected patients had 38 (18 reduced and 20 elevated) significantly altered small RNAs relative to controls (Figure 3A and Table S2). MiRNAs were the predominant species (79%) followed by misc_RNA (13%), snoRNA (2%) and rRNA (1%) (Figure 3B). CAP patients with Influenza infection had 32 significantly altered circulatory small RNAs as compared to non‐infectious control participants (Figure 3C and Table S2). Of note, 5 snRNA species (16%) were altered solely in patients with influenza infection (Figure 3D), in contrast to patients with *S pneumoniae* infection (Figure 3B). These circulating snRNA species included *RNU5A‐1*, *RNU2‐7P* and *RNU2‐33P*. 10 small non‐coding RNAs were common to both *S pneumoniae* and Influenza virus infections (Figure 3E and Table S3). Unique transcriptional alterations were also identified, with 28 and 22 significantly altered small RNAs unique to *S pneumoniae* or Influenza virus infected patients, respectively (Figure 3E and Table S3). Pathway analysis of elevated plasma miRNAs unique to *S pneumoniae* infected patients revealed an over‐representation for adherens junction, lysine degradation, bacterial invasion of the epithelial cells and endocytosis pathways (Figure 3F). Low abundance miRNAs were associated with focal adhesion and translational processing including RNA transport and mRNA surveillance pathways (Figure 3F). Pathway analysis of elevated miRNAs unique to Influenza infected patients revealed significant associations to canonical signalling pathways that included p53 signalling pathway, hippo signalling, TGF‐beta signalling pathways and MAPK signalling pathway (Figure 3G).

## 4 DISCUSSION

In this study, small non‐coding RNA levels in plasma of CAP‐associated sepsis patients were measured and compared to those of non‐infectious control participants. Relative to controls, patients presented significantly altered levels of primarily mature miRNAs in plasma. Through pathway analysis we learned, that those circulating miRNA may influence cellular mechanisms attuned to cell fate, survival and cell metabolism. Inter‐individual variation in plasma small non‐coding RNA levels in patients can be explained by differences in clinical severity and causative pathogens. We identified circulatory small RNAs that may discriminate patients with *S pneumoniae* versus Influenza (A or B) infection. Notably, snRNAs were only altered in Influenza infected patients.

In recent years, several studies have evaluated CNAs, in particular miRNAs, as potential diagnostic and prognostic biomarkers for sepsis,^8^, ^26^, ^27^, ^28^ for example, MIR30A,^29^ MIR125B2,^30^ MIR16‐2,^31^ MIR483,^31^, ^32^ MIR574,^33^ MIR210,^34^ MIR193B^31^ and MIR378C.^31^ Serum MIR126 has been proposed as a potential candidate biomarker of sepsis, with reduced levels compared to critical illness due to non‐infectious conditions (previously termed systemic inflammatory response syndrome or SIRS) or non‐infectious control participants.^35^ Notwithstanding the differences in source material, our findings showing reduced levels of MIR126 and MIRLET7I in plasma of sepsis patients (relative to controls) corroborate previous observations in serum samples.^35^ In addition to discriminating sepsis from other non‐infectious conditions or controls, studies have also reported altered expression of host leukocyte miRNAs (intracellular) comparing sepsis patients with differing causative pathogens. Recent studies reported a miRNA host response signature discriminating viral and bacterial aetiologies.^36^ Others reported decreased expression of MIR150 and MIRLET7A in patients with Gram‐negative bacilli caused urosepsis, as well as after lipopolysaccharide stimulation of a pro‐monocytic cell line.^37^ To our knowledge, our study is a benchmark evaluation of plasma non‐coding RNA levels in patients with sepsis caused by CAP with either blood culture‐positive bacterial or PCR documented viral infection. As compared to controls, patients with PCR positive influenza infection had uniquely elevated levels of snRNA. Evidence suggests snRNAs have critical roles in regulating various aspects of gene transcription, including intron splicing, polyadenylation and stability of nascent RNA.^38^ Notably, replication of influenza viruses is dependent on hijacking host cell splicing machinery, where the NS1 protein was shown to interact with several subunits of the spliceosome, including U2 and U6 snRNA classic complexes.^39^ Therefore, our findings raise the exciting possibility that plasma snRNA may constitute a novel host response signal that may differentiate viral or bacterial infections in the clinical context. Larger population‐scale studies on the diagnostic value of plasma snRNA in discriminating patients with viral or bacterial infections are certainly warranted.

CNAs, in particular miRNA, have been shown as a potential novel type of intercellular communicator.^40^ Interestingly, while intracellular RNA molecules are relatively short‐lived, extracellular miRNA have much higher stability and they are protected from degradation by endogenous RNases.^41^ The stability is conveyed by packaging into exosomes,^42^ shedding vesicles^43^ and large dense vesicles.^44^ Interactions with RNA‐binding proteins including high‐density lipoproteins,^45^ argonaute 2 (AGO2)^46^ and nucleophosmin 1 (NPM1)^47^ have been shown to stabilize cell‐free small non‐coding RNA. In so doing, the secreted microRNAs can be delivered to recipient cells where they can simultaneously regulate multiple target genes.^45^, ^48^, ^49^, ^50^, ^51^, ^52^, ^53^, ^54^, ^55^ Our findings suggest that altered levels of plasma miRNA in CAP‐associated sepsis (relative to controls) may influence various canonical signalling pathways, notably HIF‐1α signalling, hippo signalling and proteoglycan reactions. HIF‐1α signalling has been shown to influence cytokine responses both in vitro and in vivo, which is understood to be a major cog in immunometabolism wherein defects can precipitate to a state of immune paralysis (immunosuppression).^56^, ^57^ Hippo signalling is an evolutionary conserved cell developmental pathway that controls organ size by primarily regulating cell differentiation, proliferation and apoptosis.^58^ Proteoglycans represent important composite molecules of the extracellular matrix that contribute to cell adhesion, angiogenesis and in the context of cancer, metastasis and tumour progression.^59^ In particular, endocan, a proteoglycan secreted by vascular endothelium,^60^ has been reported as a candidate predictor of poor prognosis in acute respiratory distress syndrome, hospital‐acquired pneumonia, as well as in all‐cause sepsis.^61^, ^62^, ^63^

Our study has limitations. This interpretation of the study is hindered by its small sample size. Despite this limitation, genome‐wide significant differences in small non‐coding RNA were detected. The choice of source material (plasma) precluded identification of circulating small RNA cells‐of‐origin, which would enhance the functional interpretability of the small RNA profiles herein delineated.

In conclusion, we provide a comprehensive map of circulatory small non‐coding RNAs in sepsis caused by CAP relative to controls. Significantly altered circulating miRNA in patients were predicted to target various cellular signalling cascades, particularly immunometabolic, cell proliferation and survival pathways. Our findings prioritize specific circulating small RNAs for functional validation studies as well as potential biomarker studies to discriminate bacterial and viral infections in larger cohorts.

## AUTHOR CONTRIBUTION

**Hina N. Khan:** Data curation (equal); Formal analysis (equal); Writing‐original draft (equal); Writing‐review & editing (equal). **Aldo Jongejan:** Formal analysis (equal); Writing‐review & editing (equal). **Lonneke A. van Vught:** Data curation (equal); Writing‐review & editing (equal). **Janneke Horn:** Data curation (equal); Writing‐review & editing (equal). **Marcus J. Schultz:** Data curation (equal); Writing‐review & editing (equal). **Aeilko H Zwinderman:** Formal analysis (equal); Writing‐review & editing (equal). **Olaf L. Cremer:** Writing‐review & editing (equal). **Marc J. Bonten:** Writing‐review & editing (equal). **Tom van der Poll:** Conceptualization (equal); Funding acquisition (equal); Writing‐review & editing (equal). **Brendon P. Scicluna:** Conceptualization (equal); Data curation (equal); Formal analysis (equal); Supervision (equal); Writing‐original draft (equal); Writing‐review & editing (equal).

## CONFLICT OF INTEREST

The authors have no conflicts of interest to declare.

## Supporting information

Supplementary Material

Table S1

Table S2

Table S3

## Data Availability

All RNA‐sequencing data used in this study are accessible through the National Center for Biotechnology Information (NCBI) Gene Expression Omnibus (GEO) database with accession code GSE137294 (https://www.ncbi.nlm.nih.gov/geo/query/acc.cgi?acc=GSE137294).
