# Supplementary material for: The circulatory small non‐coding RNA landscape in community‐acquired pneumonia on intensive care unit admission
Source: J Cell Mol Med. 2021 Jul 17;25(16):7621–30. doi: 10.1111/jcmm.16406 (PMC8358855; doi:10.1111/jcmm.16406)
Supplement: Supplementary file 1 — Supplementary Material [file JCMM-25-7621-s003.pdf]

A

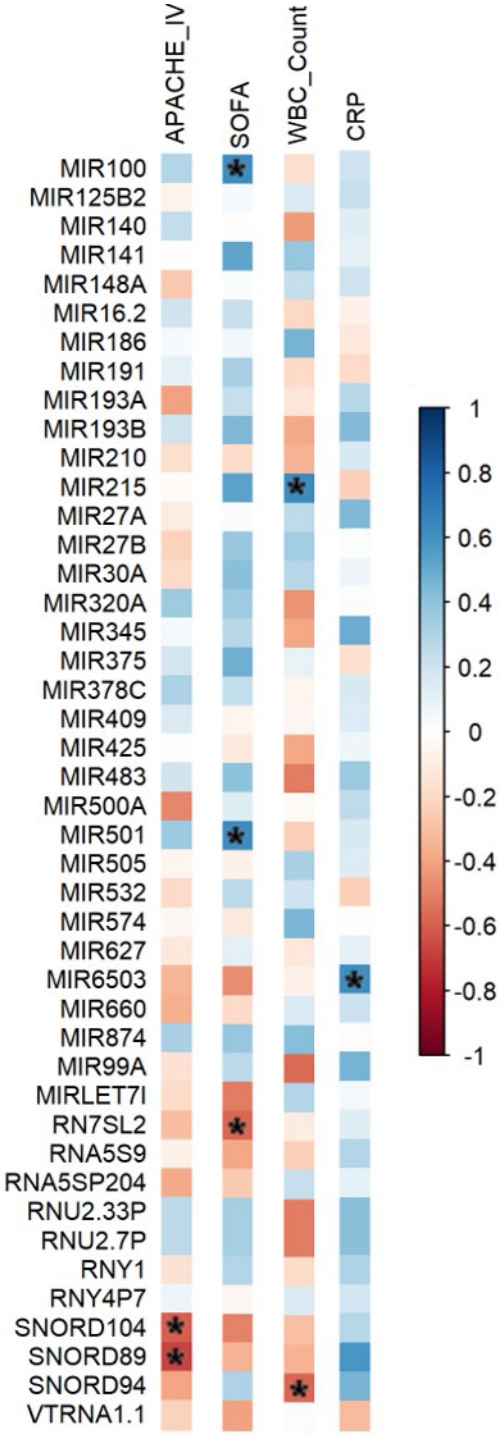

Supplementary Figure 1:
Correlation analysis of circulatory small non-coding RNA expression against clinical severity indices of patients. Acute Physiology and Chronic Health Evaluation (APACHE) IV, modified Sequential Organ Failure Assessment (mSOFA) severity scores, white blood cells(WBC) clounts and C-reactive protein (CRP).
